# Correlation Between Serum High-Sensitivity C-Reactive Protein, Tumor Necrosis Factor-Alpha, Serum Interleukin-6 and White Matter Integrity Before and After the Treatment of Drug-Naïve Patients With Major Depressive Disorder

**DOI:** 10.3389/fnins.2022.948637

**Published:** 2022-07-13

**Authors:** Liping Chen, Xiangling Zeng, Sijia Zhou, Zhiwen Gu, Jiyang Pan

**Affiliations:** ^1^Department of Psychiatry, Guangzhou First People’s Hospital, South China University of Technology, Guangzhou, China; ^2^Department of Radiology, School of Medicine, Guangzhou First People’s Hospital, South China University of Technology, Guangzhou, China; ^3^Department of Medical Imaging, Huizhou Municipal Central Hospital, Huizhou, China; ^4^Department of Psychiatry, The First Affiliated Hospital, Jinan University, Guangzhou, China

**Keywords:** major depressive disorder, white matter, inflammation, high-sensitivity C-reactive protein, diffusion tensor imaging

## Abstract

**Background:**

Previous studies have noticed that systemic inflammation may alter the integrity of white matter. However, how the levels of serum cytokine affect the integrity of white matter in major depressive disorder (MDD) patients are unclear. Our study aimed to investigate the association between the inflammatory cytokine levels and white matter microstructure in drug-naïve patients with MDD pre- and post-treatment.

**Method:**

In total, 29 MDD patients and 25 healthy controls (HC) were included in this study. Diffusion tensor imaging (DTI) was conducted in all subjects at baseline, and the MDD patients were reassessed after venlafaxine treatment, using a tract-based spatial statistics (TBSS) analysis. Morning serum interleukin-6 (IL-6), tumor necrosis factor-alpha (TNF-α), and high-sensitivity C-reactive protein (hs-CRP) concentrations in MDD patients were also measured pre- and post-treatment.

**Results:**

Significantly reduced fractional anisotropy (FA) values were found in the bilateral superior fronto-occipital fasciculus (SFO), posterior limb of the internal capsule (IC-PL), and fornix compared with the HC, and FA values in these regions in MDD patients have risen to normal levels except the bilateral SFO after treatment. The FA value of the left IC-PL was inversely correlated with the peripheral hs-CRP levels in both pre- and post-treatment MDD patients.

**Conclusion:**

Our results suggested that the white matter integrity in the left IC-PL was significantly inversely correlated with the peripheral hs-CRP levels in both pre- and post-treatment MDD patients.

## Introduction

Growing research suggests white matter fibers as key components of the brain changes in MDD patients (Korgaonkar et al., 2011; Cole et al., 2012; de Diego-Adelino et al., 2014; Korgaonkar et al., 2014). Diffusion tensor imaging (DTI) is a method to assess the abnormalities of white matter tracts that could help study pathophysiology mechanisms for depression. Fractional anisotropy (FA) is recognized as one of the quantitative parameters derived from DTI, which is more sensitive to pathological conditions and can reflect microstructural abnormalities of white matter integrity (Jenkins et al., 2016). Decreased FA is known to indicate the disruption of white matter, lowered myelination, and/or reduced axonal density (Beaulieu, 2002). Previous studies have reported reduced FA values in various white matter areas among MDD patients in comparison with individuals (Korgaonkar et al., 2011; Guo et al., 2012; Han et al., 2014; Xia et al., 2018). However, the pathogenesis and the treatment response of WM alteration in MDD have not been well understood.

Current evidence indicated that inflammatory processes play a key role in the pathogenesis of MDD (Dowlati et al., 2010; Leonard and Maes, 2012; Haapakoski et al., 2015) and response to treatment (Vollmar et al., 2009; Fornaro et al., 2011; Valkanova et al., 2013). Higher concentrations of inflammation in MDD patients were detected in comparison with healthy controls. However, the specific inflammatory markers being measured are crucial, i.e., tumor necrosis factor (TNF)-α, interleukin (IL)-6, and C-reactive protein (CRP) (Howren et al., 2009; Dowlati et al., 2010; Hiles et al., 2012; Valkanova et al., 2013; Liu et al., 2016). Prior study has found that microstructural changes in white matter fibers in emotional and cognitive approaches are linked with the psychopathology of bipolar disorder (Benedetti et al., 2011). However, this topic has not received much attention among drug-naïve patients with MDD. A cross-sectional study found that the reduced FA values of the bilateral inferior fronto-occipital fasciculus and corpus-callosum in the early stage of MDD correlated with IL-1β levels (Sugimoto et al., 2018). Another community study reported that higher C-reactive protein level in mid-life was correlated with the decreased FA value on brain DTI in later years (Walker et al., 2017). These growing pieces of evidence suggest that inflammatory markers might alter WM microstructure in MDD. We hypothesized that elevated inflammatory cytokines may alter the microstructure of WM tracts in drug-naïve MDD patients, and reduced inflammatory cytokines may help improve the microstructural abnormalities of WM integrity after antidepressant treatment. We aimed to explore the relationship between the peripheral hs-CRP, IL-6, and TNF-α, levels and WM microstructure in drug-naïve patients with MDD pre- and post-treatment with venlafaxine.

## Materials and Methods

### Participants

Our MDD patients aged between 18 and 50 years were enrolled at the outpatient clinical sites of the Department of Psychiatry, Guangzhou First People’s Hospital, South China University of Technology, China, between October 2017 and October 2018. The healthy controls were included *via* advertisements. Accordingly, 29 drug-naïve patients with MDD and 25 age, gender, BMI, and education level paired with healthy controls were recruited (Table 1). All participants in our study went through the Structured Clinical Interview for DSM-IV-TR Axis I Disorders-Patient Edition (SCID-I/P) by two experienced psychiatrists. Our patients met the criteria for MDD but no other mental disorders were found, and all healthy controls were confirmed no history of any major DSM-IV axis I disorder or a family history of major psychiatric illnesses according to DSM-IV-TR. Drug-naïve patients with MDD were chosen (MDD patients who have never taken any kind of antidepressants). The 17-item Hamilton Depression Rating Scale (HAMD-17) (Hamilton, 1967) was implemented to discover the severity of depression. All recruited MDD patients had scored ≥ 17 on the 17-item HAMD. Participants with any moderate or severe physical illness (diabetes, hypertension, dyslipidemia, and infectious disorders), drug and alcohol abuse or dependence, or any neurological disease, and the existence of other mental disorders were excluded. Those MDD patients with high suicide risk or BMI > 25 were also excluded. None of the subjects had a history of childhood trauma, no major life events within 1 year were found, and no medications that influence the immune system (steroids, aspirin, or non-steroidal anti-inflammatory drugs) were used. The drug-naïve MDD patients were treated with venlafaxine at 150–225 mg daily plus benzodiazepines if needed. The treatment period was 8 weeks. An experienced neuroradiologist evaluated all of the MRI scans to make sure that the subjects didn’t have a severe brain injury. This study was approved by the Hospital’s ethics committee. Written informed consent was obtained from all participants before the beginning of the study.

**TABLE 1:** Demographic and clinical data for MDD participants and healthy controls.

Groups Clinical variables	MDD patients (mean ± *SD*)	Healthy controls (mean ± *SD*) (mean ± *SD*) S
Sex (M/F)	5/24	2/23
Age (years)	34.48 ± 14.83	35.00 ± 13.91
Education (years)	12.72 ± 3.81	14.68 ± 3.96
Duration (months)	36.68 ± 30.81	-
Depressive episodes	1.76 ± 0.83	-
Smoke(Y/N)	2/29	0/25
BMI	21.52 ± 3.44	21.61 ± 2.56
HAMD-17		
Baseline	22.79 ± 4.84**	3.07 ± 1.21
After treatment	5.86 ± 2.93^##^	-
Hs-CRP (mg/l)		
Baseline	0.88 ± 1.07^#^	1.11 ± 1.07
After treatment	0.36 ± 0.51**	-
TNF-α(pg/ml)		
Baseline	48.45 ± 29.29^##^	41.35 ± 15.86
After treatment	16.65 ± 8.08**	-
IL-6 (pg/ml)		
Baseline	6.65 ± 4.62^#^	7.89 ± 3.84
After treatment	4.44 ± 2.81*	-

Values are represented as mean ± SD. *p < 0.05, **p < 0.01, pretreatment major depressive disorder vs. controls or post-treatment major depressive disorder vs. controls. ^#^p < 0.05, ^##^p < 0.01, pretreatment major depressive disorder vs. post-treatment major depressive disorder. SD, standard deviation; MDD, major depression disorders; HAMD-17, 17-item Hamilton Rating Scale for Depression; hs-CRP, high-sensitivity C-reactive protein; TNF-α, tumor necrosis factor (TNF)-α; IL-6, interleukin (IL)-6.

### Cytokine Measurements

We collected peripheral venous blood samples from 29 MDD participants and 25 healthy individuals on admission between 8 and 9 a.m., overnight, and 30 min after waking and resting. MDD patients were reassessed after treatment with venlafaxine. All participants slept overnight in the lounge of the hospital before morning cytokine measurements. The plasma was isolated and stored at –80°C. Serum concentrations for high-sensitivity CRP levels (mg/L) were measured by immuneturbidimetric assay, immediately after taking the samples with commercial kits (Roche Diagnostic, Switzerland) on Roche C702 automatic analyzer. The normal reference range is < 3 mg/L. Serum levels of TNF-α and IL-6 were measured using the enzyme-linked immunosorbent assay (ELISA), immediately after the collection of samples by commercial kits (RayBiotech, United States). Mean intra- and interassay coefficients of variation (CV) were < 10%.

### Image Data Acquisition

Both MRI and DTI were performed on a clinical 3.0-T Siemens MAGNETOM Verio scanner (Siemens, Erlangen, Germany), using a twelve-channel head and neck coil. Scan parameters were TR/TE = 8,700 ms/102 ms, FOV = 230 mm × 230 mm, slice thickness = 2.5 mm, number of excitations = 2, and spatial resolution = 2.5 × 2.5 × 2.5 mm. Diffusion gradients (*b* = 2,000 s/mm^2^) were applied along 99 non-collinear directions simultaneously.

### Imaging Analysis

The distortion of the diffusion tensor images was corrected by eddy current correction using FRIB software library (FSL) v4.1.0, Oxford.^1^ Voxel-wise statistical analysis of FA, mean diffusivity (MD), radial diffusivity (RD), and axial diffusivity (AD) values was performed using TBSS version 1.1. Images of diffusion indicators were converted from native space to the standard space of 1 mm^3^ Montreal Neurological Institute (MNI). We used FMRIB’s Non-Linear Image Registration Tool (FNIRT, part of FSL) to non-linearly register the participant’s FA image into the FMRIB58 FA template. FA data of each subject were projected to the mean FA skeleton built from averaging adjusted individual FA images. WM tracts were represented by the FA skeleton, and the mean skeleton at FA was built with the threshold of 0.2 (Smith et al., 2006). FA images were adjusted with an 8-mm full width at half maximum Gaussian filter.

### Statistical Analysis

All statistical analyses were done using the Statistical Package for Social Sciences, version 22.0 (SPSS, Chicago, IL). We applied the chi-square and independent samples *t*-test to analyze statistics between MDD and HC groups concerning demographic and clinical variables. FA, MD, AD, and RD values and cytokine levels of patients before and after treatment were compared with a paired sample *t*-test. The relationship between serum cytokine concentrations and the FA values was evaluated with a single-group mean value with a partial correlation test controlling for age, education, and duration. Two-tailed with alpha set to 0.05 was used for all analyses. We applied multiple comparisons of statistical results in the randomized tool of FSL using the method of TFCE (threshold-free cluster enhancement). We indicated a statistically significant correlation between the groups after multiple comparisons were made at the cluster level after adjustment for family-wise error (FWE) and considered values of *p* < 0.05 and > 20 voxels. We set the number of permutations in all voxel-wise analyses at 5,000. In all voxel analyses, age and gender were included in the statistical model as non-sense variables using the TBSS.

## Results

### Participants

The demographic data of all participants are presented in Table 1. No statistically significant differences were found between the patient and HC groups in age, gender, BMI, and education level at baseline. Twenty of the patients had their first episode, and all patients were free of drug treatment (including anxiolytics) in the study; 29 MDD patients finished the serum cytokine levels collection and DTI scans at baseline, and one patient’s DTI result was unavailable; 8 patients dropped out of the study, where 5 patients could not tolerate the side effects of venlafaxine and 3 patients dropped out of the study due to other reasons. Hence, 20 MDD patients were reevaluated after 8 weeks of venlafaxine treatment. The severity of depression decreased significantly after treatment. No significant distinctions were observed in all cytokines measured between the MDD and HC. However, peripheral levels of hs-CRP, TNF-α, and IL-6 in MDD patients were reduced, lower than that in HC after venlafaxine treatment.

### Comparison of WM Integrity Change Between Major Depressive Disorder Patients and Healthy Controls

**FIGURE 1:**
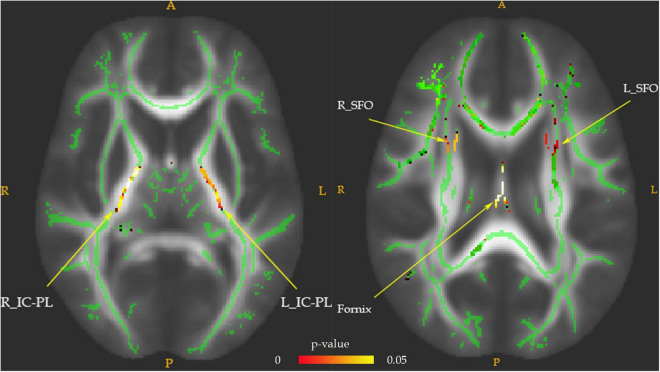
The FA values comparison between pretreatment MDD and HC groups. Lower FA in MDD patients (*n* = 28) vs. HC (*n* = 25) in the fornix tract, bilateral superior fronto-occipital fasciculus (SFO), and bilateral posterior limb of the internal capsule (IC-PL). The white matter FA values of MDD patients were significantly lower than that of the control group in axial slices, as demonstrated in red-green (FWE-corrected *p* < 0.05, cluster > 20).

**FIGURE 2:**
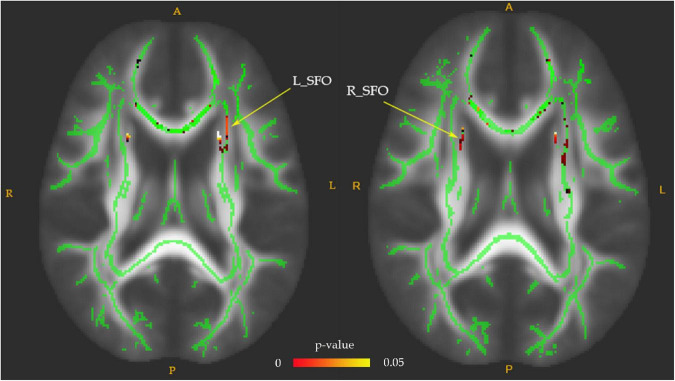
The FA values comparison between post-treatment MDD and HC groups. The decreased FA values of the bilateral superior fronto-occipital fasciculus (SFO) in the post-treatment MDD patients were still significantly lower than that of HC, as indicated in red-green (FWE-corrected *p* < 0.05, cluster > 20).

Fractional anisotropy reductions (FWE-corrected, *p* < 0.05) in the bilateral posterior limb of the internal capsule, superior fronto-occipital fasciculus (SFO), and fornix were found in the drug-naïve MDD patients compared with HC at baseline (Figure 1). Cluster labels and significant MNI space are reported in Table 2. No significant differences were observed for the AD, RD, and MD. After the treatment, FA values in these regions rose to normal levels except for the bilateral SFO. The reduced FA values in the bilateral SFO were still lower than that in the HC. No region in post-treatment MDD showed a higher FA value than that in the HC (Figure 2).

**TABLE 2:** The FA values comparison between groups at baseline (control group > MDD group).

Anatomical regions	MNI coordinates, mm			Number of voxels	*p*
	
	*X*	*Y*	*Z*		
L_SFO	111	113	80	45	0.021*
R_SFO	69	112	80	54	0.023*
L_IC-PL	112	133	93	882	0.014*
R_IC-PL	69	136	93	880	0.048*
Fornix	88	111	90	162	0.017*

*p < 0.05, FWE-corrected. MNI, Montreal Neurological Institute. FA, fractional anisotropy; MDD, major depressive disorder; L_SFO, the left superior fronto-occipital fasciculus; R_SFO, the right superior fronto-occipital fasciculus; L_IC-PL, the left posterior limb of the internal capsule; R_IC-PL, the right posterior limb of the internal capsule; Fornix, the fornix tract.

### Correlation Between Peripheral Inflammatory Cytokine Levels and WM Integrity

**FIGURE 3:**
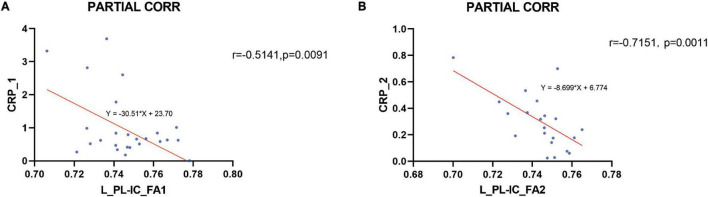
Correlation between the peripheral hs-CRP levels and the FA in the left posterior limb of the internal capsule (L_PL-IC) before and after treatment. An inverse correlation between the FA values in the left posterior limb of the internal capsule (L_PL-IC) and peripheral hs-CRP levels in both before and after treatment patients was found when controlling for age, duration, and education by partial correlation (**A** pretreatment, **B** post-treatment).

The FA values of the left PL-IC and genu of the corpus callosum revealed significant inverse correlations between peripheral hs-CRP levels in pretreatment MDD patients after controlling age, duration, and year of education. The decreased FA in the left PL-IC rose to normal levels and was also significantly negatively correlated with hs-CRP levels after treatment in post-treatment MDD (Figure 3). No areas presented a significant connection between other serum cytokine levels and FA values in drug-naïve MDD patients before and after treatment with venlafaxine. There were no areas that showed a significant correlation between peripheral cytokine levels and FA in HC. We found an inverse correlation between the FA values in the left PL-IC and peripheral hs-CRP levels in both pre- and post-treatment patients when controlling for age, duration, and education by partial correlation.

## Discussion

In this study, decreased FA values in the bilateral PL-IC, SFO, and fornix in pretreatment MDD patients rose to normal levels to some extent after treatment. In addition, FA values in the left PL-IC in MDD patients were significantly inversely associated with the peripheral hs-CRP levels before and after treatment. These values were also approved by the partial correlation analysis, after controlling age, education, disease course, and other confounding factors.

No statistically significant differences in the concentration of inflammatory cytokines were noticed between the MDD patients and HC at baseline. However, after the treatment, peripheral hs-CRP, IL-6, and TNF-αlevels in MDD patients were reduced obviously and even lower than that in HC. Previous studies showed that low-grade inflammation is a key characteristic of MDD with higher concentrations of inflammatory markers such as C-reactive protein (CRP) and TNF-α and IL-6 levels (Duivis et al., 2013; Valkanova et al., 2013; Dahl et al., 2014). Our negative results for the cytokines levels are also consistent with the results of Meier et al. (2016) and Sugimoto et al. (2018) with similar BMI scores in the MDD and HC groups and our strict inclusion criteria.

In our study, 74.1% of patients achieved clinical remission after 8 weeks of venlafaxine treatment. With significantly improved depression symptoms, the post-treatment hs-CRP, TNF-α, and IL-6 levels in the drug naïve patients with MDD were lower than that in the HC. Similarly, Li et al. (2013) pointed out that serum TNF-α levels were significantly reduced after venlafaxine treatment in the drug naïve and first-episode patients with MDD. Previous studies also reported that antidepressants may decrease peripheral inflammation (Kohler et al., 2018), and antidepressant response is modulated by neuroinflammatory pathways (Miller et al., 2017). We speculate that the decrease in plasma hs-CRP, TNF-α, and IL-6 levels is due to the pharmacological action of venlafaxine. Peripheral cytokines level in the acute phase of MDD patients might be over-corrected by venlafaxine, and whether it reconstructs to normal level will need further research.

We should note that high peripheral hs-CRP levels were significantly inversely associated with FA values of the left IC-PL in both pre- and post-treatment MDD patients in this study. Peripheral cytokines are considered to have central effects, either through the transmission of signals by the vagus nerve or through crossing the blood–brain barrier (Dantzer et al., 2008). CRP and other inflammatory cytokines in MDD peripheral blood may mirror the inflammatory activity of CNS (Felger et al., 2020). Serum CRP is a crucial flag of peripheral inflammation and plays a key role in the occurrence, development, and treatment of MDD (Au et al., 2015; Cattaneo et al., 2015; Liu et al., 2020). The elevation of hsCRP levels generally determines low-grade inflammation (Howren et al., 2009; Duivis et al., 2013; Valkanova et al., 2013; Dahl et al., 2014). We inferred that white matter fibers in MDD patients might be more sensitive to hsCRP than healthy controls, and MDD patients with normal hsCRP levels in the early stage might have already undergone the microstructural alteration in the WM tracts. A previous study has demonstrated that chronic social stress can promote serum cytokines to cross the BBB or alter the integrity of the BBB by promoting the reduction of endothelial tight junction protein Claudin-5, suggesting that inflammatory cytokines may affect brain parenchyma more easily in participants with MDD than in healthy individuals (Menard et al., 2017). Peripheral serum inflammation cytokines are correlated with both the structural and functional abnormalities of the brain in MDD patients (Frodl and Amico, 2014). For example, according to a study by Frodl et al. (2012), the elevation of CRP and IL-6 levels was significantly inversely correlated with decreased bilateral hippocampal volume in MDD, which suggests that increasing inflammatory cytokines might have an important part in the neuroplasticity-neurotoxicity cascade. It was pointed out that abnormalities of white matter microstructure in systems crucial for the cognitive and emotional procedures may be related to the neuropathological mechanism of BD (Benedetti et al., 2011). However, less research has been conducted to investigate this topic. In a previous DTI study, a higher C-reactive protein level in midlife was associated with significant FA reductions in the brain in late life (Walker et al., 2017). Furthermore, another meta-analysis predicted that the high level of systemic inflammation among MDD patients would change the integrity of white matter, which influences the progress of the disorder into a chronic form (Goldsmith et al., 2016). Our findings are consistent with the previous evidence that systemic inflammation in MDD patients was correlated with microstructural changes in white matter tracts. Furthermore, we also found that the neuroinflammatory state was associated with microstructural alterations in white matter fibers after successful antidepressant treatment, which provides the first evidence to support the preliminary relationship between peripheral hs-CRP levels and white matter changes. The injury of white matter integrity can be reversed after successful antidepressant treatment in our MDD patients, which could be supported by some previous research. Wohleb et al. (2011) suggested that therapeutic interventions marking stress-related neuronal differences in the hippocampus would be helpful for MDD patients. This evidence supports our speculation that relatively high levels of systemic inflammation may lead to WM injury in the MDD brain, and this injury can be restored by successful antidepressant treatment to some extent.

Several other inflammatory cytokines, such as TNF-α and IL-6, are thought to have a more significant effect on neurological function. However, we only found that relatively high levels of hsCRP were negatively correlated with the microstructural alterations in the white matter in MDD patients. It could be due to IL-6 and TNF-α being also sensitive to stress, and we excluded patients with recent and previous major life event trauma and childhood trauma but did not evaluate their short-term stress levels. Evidence suggests that CRP levels are relatively constant even in the absence of disorder (Kluft and de Maat, 2001).

In terms of regional specificity, our findings are also consistent with several previous DTI data that identified WM-reduced FA values in SFO (Phillips et al., 2003), posterior limb of the internal capsule (IC-PL) (Alves et al., 2012; Ghazi Sherbaf et al., 2018), and fornix (Hoogenboom et al., 2014) of MDD patients. However, no significant changes in FA between patients with MDD and healthy individuals were reported by a large sample study (Choi et al., 2014). It is possible that studying a wide range of patients with different demographic characteristics (Wise et al., 2014), episodes or duration of illness (Wise et al., 2014), and antidepressants (Khalaf et al., 2015; Zhang et al., 2015) results in inconsistent findings. In addition, myelination and WM volume have been addressed to alter with age (Giorgio et al., 2010; Belov and Pshuk, 2020). Thus, in our study, examinations of the integrity of white matter sections in younger patients with drug-naïve MDD before and after treatment are critical to understanding the pathophysiology of this disease (Cullen et al., 2010).

It is crucial to point out that the peripheral hs-CRP levels were only specifically associated with the FA values in the left PL-IC in MDD patients before and after treatment. Our results were similar to large-scale research by Green et al. (2020), suggesting a correlation between DNAmCRP and FA values in the external and internal capsules. The internal capsule is a major subcortical combining structure linking cortical-subcortical regions, which is located between the caudate nucleus, the dorsal thalamus, and the legume nucleus. Previous research suggested that people with depression have damaged cortical-subcortical neural circuits (Sexton et al., 2009), mainly involving abnormalities in white matter tracts in different regions including emotional regulation (Liao et al., 2013), cognition (Austin et al., 2001), or reward circuits (Bracht et al., 2015).

The limbic-thalamic-frontal lobe circuit is important in the neuropathology arrangement of depression. The posterior limb of bilateral internal capsules is a crucial structure of this processing network (Wakana et al., 2004); impacts cognitive, emotional, and behavioral functions; and its impairment is associated with cognitive-related, primary, or post-stroke depression (Xiao et al., 2015). We found that only FA values in the left PL-IC were significantly inversely correlated with high serum hs-CRP levels in MDD pre- and post-treatment. It might be supported by some previous studies suggesting that in MDD, cortico-limbic-striatal findings are left-lateralized (Strawn et al., 2014; Yang et al., 2015; Niu et al., 2017).

This is the first longitudinal study to demonstrate the relationship between serum cytokine levels and white matter alterations in drug-naïve MDD patients before and after antidepressant treatment. We provide the first evidence to discover the associations between peripheral hs-CRP levels and WM alterations. The advantages of our study lie in the confounding factors such as age, course of the disease, medication status, comorbidities, and education, BMI, and lifestyle that were strictly controlled, which were relatively rare in similar DTI studies (Benedetti et al., 2016; Sugimoto et al., 2018). Moreover, we also used 99 non-collinear diffusion-sensitive gradient directions in the DTI scan, which can more accurately measure the changes of the WM fiber and improve the reliability of the results (Jones, 2004). However, some potential limitations of the study could be taken into consideration. The major problem in this study is the limited sample size that might decrease the power of our results due to our rigorous exclusion criteria, but the relatively small sample size is very common in similar imaging studies (Wang et al., 2012; Benedetti et al., 2016; Sugimoto et al., 2018), especially for longitudinal studies (Haroon et al., 2014; Lin et al., 2016; Tsuchiyagaito et al., 2021); the statistical power of this sample is generally recognized. The relatively small sample size of this study is for the following two reasons: First, it was difficult to find drug-naïve MDD with such strict inclusion criteria and second, the patients enrolled in our study also had a relatively high loss rate after 8 weeks of treatment with venlafaxine. Future studies should strengthen the follow-up of the patients’ medication status and further improve the patients’ compliance with the treatment. It should be noted that all MDD patients’ hs-CRP levels were less than 3 mg/L at baseline, and most of them achieved clinical remission after antidepressant treatment; thus, our results could only represent the MDD patients who respond to the treatment but not the resistant patients. Therefore, a larger group size with different characteristics of MDD is required to discover the correlation between serum hS-CRP levels and WM changes. Furthermore, to observe the causal associations between peripheral hs-CRP levels and WM alterations in MDD, follow-up measures of the healthy control group should be included in our further study. Moreover, to make a more accurate MDD diagnosis, machine learning-based analysis (Li et al., 2020) and graph theory-based analysis (Xu et al., 2020) are also considered in our future work.

In conclusion, significantly increased FA values were found in the bilateral SFO, PL-IC, and fornix compared with the pretreatment values among early stage MDD patients. The FA values in the left PL-IC areas were inversely correlated with the peripheral hs-CRP levels in both pre- and post-treatment patients. Our results suggested that the changes in white matter integrity in the left PL-IC are associated with serum hs-CRP levels before and after antidepressant treatment.

## Data Availability Statement

The raw data supporting the conclusions of this article will be made available by the authors, without undue reservation.

## Ethics Statement

The studies involving human participants were reviewed and approved by the Ethics Committee of Guangzhou First People’s Hospital. The patients/participants provided their written informed consent to participate in this study.

## Author Contributions

LC contributed to the design of the research and the writing of the manuscript. All authors participated in the data collection in this manuscript.

## Conflict of Interest

The authors declare that the research was conducted in the absence of any commercial or financial relationships that could be construed as a potential conflict of interest.

## Publisher’s Note

All claims expressed in this article are solely those of the authors and do not necessarily represent those of their affiliated organizations, or those of the publisher, the editors and the reviewers. Any product that may be evaluated in this article, or claim that may be made by its manufacturer, is not guaranteed or endorsed by the publisher.

## Footnotes

1
https://www.fmrib.ox.ac.uk/fsl
